# The Segond fracture occurs at the site of lowest sub‐entheseal trabecular bone volume fraction on the tibial plateau

**DOI:** 10.1111/joa.13282

**Published:** 2020-08-08

**Authors:** William Mullins, Gavin E. Jarvis, Daniel Oluboyede, Linda Skingle, Ken Poole, Tom Turmezei, Cecilia Brassett

**Affiliations:** ^1^ Human Anatomy Centre Anatomy Building Department of Physiology, Development and Neuroscience University of Cambridge Cambridge UK; ^2^ Department of Medicine Cambridge NIHR Biomedical Research Centre University of Cambridge Cambridge UK; ^3^ Norfolk and Norwich University Hospitals NHS Trust Norwich UK

**Keywords:** anterolateral ligament, avulsion, BV/TV, microCT, microView, segond fracture, tibial Plateau, trabecular bone volume fraction

## Abstract

In a series of human cadaveric experiments, Dr. Paul Segond first described the avulsion injury occurring at the anterolateral tibial plateau that later took his name. The fracture is thought to arise as a consequence of excessive tibia internal rotation which often also elicits damage to other connective tissue of the knee. The exact mechanism behind the avulsion is, however, unclear. A number of ligamentous structures have been proposed in separate studies to insert into the Segond fragment. Suggestions include the iliotibial band (ITB), biceps femoris and the controversial ‘anterolateral ligament’ (ALL). Despite increasing knowledge of tibial plateau bony microarchitecture in both healthy and disease states, no studies have yet, to our knowledge, considered the role of tibial sub‐entheseal bone structure in pathogenesis of the Segond fracture. The goal of this study was thus to elucidate the differences in trabecular properties at regions across the tibial plateau in order to provide an explanation for the susceptibility of the anterolateral region to avulsion injury. Twenty human tibial plateaus from cadaveric donors were dissected and imaged using a Nikon‐XTH225‐μCT scanner with <80 μm isotropic voxel size. Scans were reconstructed using MicroView 3D Image Viewer and Analysis Tool. Subsequent virtual biopsy at ten anatomically defined regions of interest (ROI) generated estimates of bone volume fraction (‘bone volume divided by total volume’ (BV/TV)). The overall mean BV/TV value across all 20 tibiae and all 10 ROIs was 0.271. Univariate repeated‐measurements ANOVA demonstrated that BV/TV values differed between ROIs. BV/TV values at the Segond site (Sα, Sβ or Sγ) were lower than all other ROIs at 0.195, 0.192 and 0.193, respectively. This suggests that, notwithstanding inter‐ and intra‐specimen variation, the Segond site tends to have a lower trabecular bone volume fraction than entheseal sites elsewhere on the tibia. Since BV/TV correlates with tensile and torsional strength, the lower BV/TV at the Segond site could equate to a region of local weakness in certain individuals which predisposes them to an avulsion injury following the application of force from excessive internal rotation. The low BV/TV recorded at the Segond site also challenges the idea that the fracture occurs due to pull from a discrete ‘anterolateral ligament’, as the tension exerted focally would be expected to elicit a hypertrophic response in line with Frost's Mechanostat hypothesis. Our data would instead agree with the aforementioned reports of the fibrous band at the Segond site being part of a broader insertion of an ‘anterolateral complex’.

## Graphical abstract

MicroCT data from cadaveric tibiae demonstrates that trabecular bone volume fraction at the site of the Segond fracture is significantly lower than other entheseal sites across the tibial plateau. This may equate to a region of local weakness which can predispose certain individuals to the avulsion injury following the application of excessive tibial internal rotation.

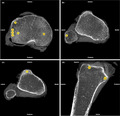

## 1 INTRODUCTION

### 1.1 The Segond fracture

In a series of human cadaveric experiments, Dr. Paul Segond (1879) first described the avulsion injury occurring at the anterolateral tibial plateau that later took his name. The Segond fracture commonly occurs alongside local soft tissue injury; studies have shown 75%–100% of injuries result in anterior cruciate ligament (ACL) tear and 66%–70% result in meniscal damage as a consequence of the trauma (Dietz *et al*. 1986; Goldman *et al*. 1988). It has been suggested that this is due to soft tissue structures of the anterolateral region being placed under a comparable degree of strain to intracapsular ligaments by tibial internal rotation (Dodds *et al*. 2014). Damage to these anterolateral soft tissue structures have been shown to elicit a positive pivot shift phenomenon—a finding classically indicative of ACL tear (Hughston *et al*. 1976; Bull *et al*. 1999)—suggesting this tissue is involved in maintaining similar axes of knee stability as the ACL. Moreover, anterolateral structures have been suggest to have a more important role in resisting internal rotation because of the larger moment arm they carry compared to the more centrally located ACL (Amis 2017).

Almost a century after Segond's original reports of a ‘*pearly*,*resistant*,*articular fibrous band’* (Segond 1879, p. 14) that was placed under strain by the same internal rotation forces that are resisted by the ACL, Kaplan (1958) proposed that deep fibres of the iliotibial band (ITB) insert into the Segond fragment. Later groups suggested other insertions, including part of the short head of biceps femoris (Terry and LaPrade 1996) and an extension of the lateral capsular ligament (Johnson 1979; Woods *et al*. 1979). These observations underlie the classification of the injury as an avulsion, insofar as the mechanism involves a soft tissue structure pulling on the bone at the site of insertion. More detailed analysis later suggested that the fibres which insert into the Segond fragment may be considered—functionally or anatomically—part of a distinct ligamentous structure: the ‘anterolateral ligament’ (ALL). Comparison of radiological data from patients with a possible Segond fracture with cadaveric reports of the ALL's tibial insertion demonstrated that the avulsion occurred at the exact site of ALL insertion (Claes *et al*. 2014). Biomechanical analysis has shown that, like the ACL, the ALL is an important stabiliser of internal rotation, and may play a more important role in stability during knee flexion (Parsons *et al*. 2015).

There is a lack of consensus surrounding the incidence of the ALL in adult knees, with some authors arguing for inexistence whilst others claiming a presence in 100% of knees (Ariel de Lima *et al*. 2019). Arguments against include MRI data has suggested that the ALL is inseparable from neighbouring lateral collateral ligament (LCL) and ITB (Porrino *et al*. 2015). It follows that the Segond fragment could, therefore, receive insertion from one of several closely opposed ligamentous and capsular attachments within the ‘anterolateral complex’ (Shaikh *et al*. 2017). Knowledge of the anatomy of this region is important for characterising the mechanisms of traumatic internal structural derangement and to help guide anterolateral capsule repair—an intervention which has been shown to restore rotational stability and correct pivot shift (Ferretti *et al*. 2017b).

### 1.2 MicroCT and virtual biopsy

Micro‐computed tomography (μCT) is an imaging modality which is limited to scans of smaller scale specimens than a typical clinical CT scanner. It is, however, able to do so at a much higher resolution with a pixel size in the order of 10 s compared to 100 s of microns. The detail of the acquired image allows for repeated ‘virtual biopsy’ of a specimen in a non‐destructive manner, while the richness of information also allows sampling of the image data volume at locations that can be selected from multiple orthogonal viewing planes. μCT has advantages over dual‐energy X‐ray absorptiometry (DXA) for assessment of bone mineral density (BMD) due to its ability to incorporate three dimensions in the reconstructed imaging in addition to the superior resolution. It is therefore used as a means of conferring validity to novel DXA techniques attempting to mimic the resolution of other imaging modalities (Briggs *et al*. 2010). DXA remains the clinical modality of choice for assessment of BMD because of the lower radiation dose, relatively low cost and ability to scan larger specimens (Kleerekoper and Nelson 1997). μCT is instead currently restricted—in human imaging research—to analysis of ex vivo specimens.

Variations in apparent bone trabecular volume fraction are related by a power‐law function to bone tensile and torsional strength (Kaplan *et al*. 1985; Sarin *et al*. 1999). Trabecular bone has been shown to have a significantly lower tensile strength compared to compression strength, explaining why the force of injury in avulsion fractures is typically much lower than that seen in other types of fracture (Kaplan *et al*. 1985). As a precedent, μCT has been used to quantify the correlation between trabecular bone volume fraction and strength parameters—namely, Young's modulus, yield stress and ultimate stress—in cadaveric tibiae (Lancianese *et al*. 2008). Trabecular bone volume fraction (bone volume over total volume, BV/TV, expressed in %) recorded using μCT has also been used as a means of predicting strength and stiffness in both normal and pathological trabecular bone (Nazarian *et al*. 2008).

### 1.3 Gaps in the field and aims of the current study

Despite increasing knowledge of tibial plateau bony microarchitecture in both healthy and disease states, no studies have yet, to our knowledge, considered the role of tibial sub‐entheseal bone structure in pathogenesis of the Segond fracture. The goal of this study was thus to elucidate the differences in trabecular properties according to μCT analysis at regions across the tibial plateau and quantify the relative bone densities underlying each enthesis. When referencing entheseal sites, we intend to discuss each as a functional organ—including adjacent trabecular bone structure in addition to the cortex which receives the insertion. We hypothesised that BV/TV at the Segond site was lower than other entheses across the plateau, explaining the propensity for avulsion.

## 2 MATERIALS AND METHODS

### 2.1 Dissection

Lower limb specimens were randomly selected for dissection from human cadavers. All donors had provided written consent before decease for their bodies to be used for anatomical research, in compliance with the Human Tissue Act 2004. Specimens with evidence of overt knee trauma, surgery or degenerative joint disease were excluded. We further eliminated specimens whose records stated the cause of death was from breast, prostate or lung cancer, as these are the most common cancers which seed bony metastases (Mundy 2002; Svensson *et al*. 2017). Fifteen female (age range, 72–99; mean(*SD*) age, 87.2(8.4) years) and five male (age range, 82–93; mean(*SD*) age 87.4(5.3) years) donors passed the initial screening and were thereby included in the study. A standardised dissection procedure was used for each specimen, involving removal of skin and soft tissues, as well as disarticulation of the knee joint. The isolated tibial plateaus remained connected to the adjacent fibulae by their associated ligaments. Both were cut to around 10 cm in length such that they would fit within the apparatus for loading into the μCT scanner. Tendons were left in place to act as reference points for later virtual biopsy.

### 2.2 MicroCT scanning

Specimens were packed individually into a polystyrene holding container such that they would stand upright independently and remain stationary during rotation of the platform within the scanner. The holding containers were loaded into and imaged using a Nikon XTH225 μCT scanner (Nikon Metrology UK Ltd.), with <80 μm isotropic voxel size. The scan time for each sample was approximately 25 min. DICOM imaging output was exported for viewing and analysis.

### 2.3 Virtual biopsy

**FIGURE 1:**
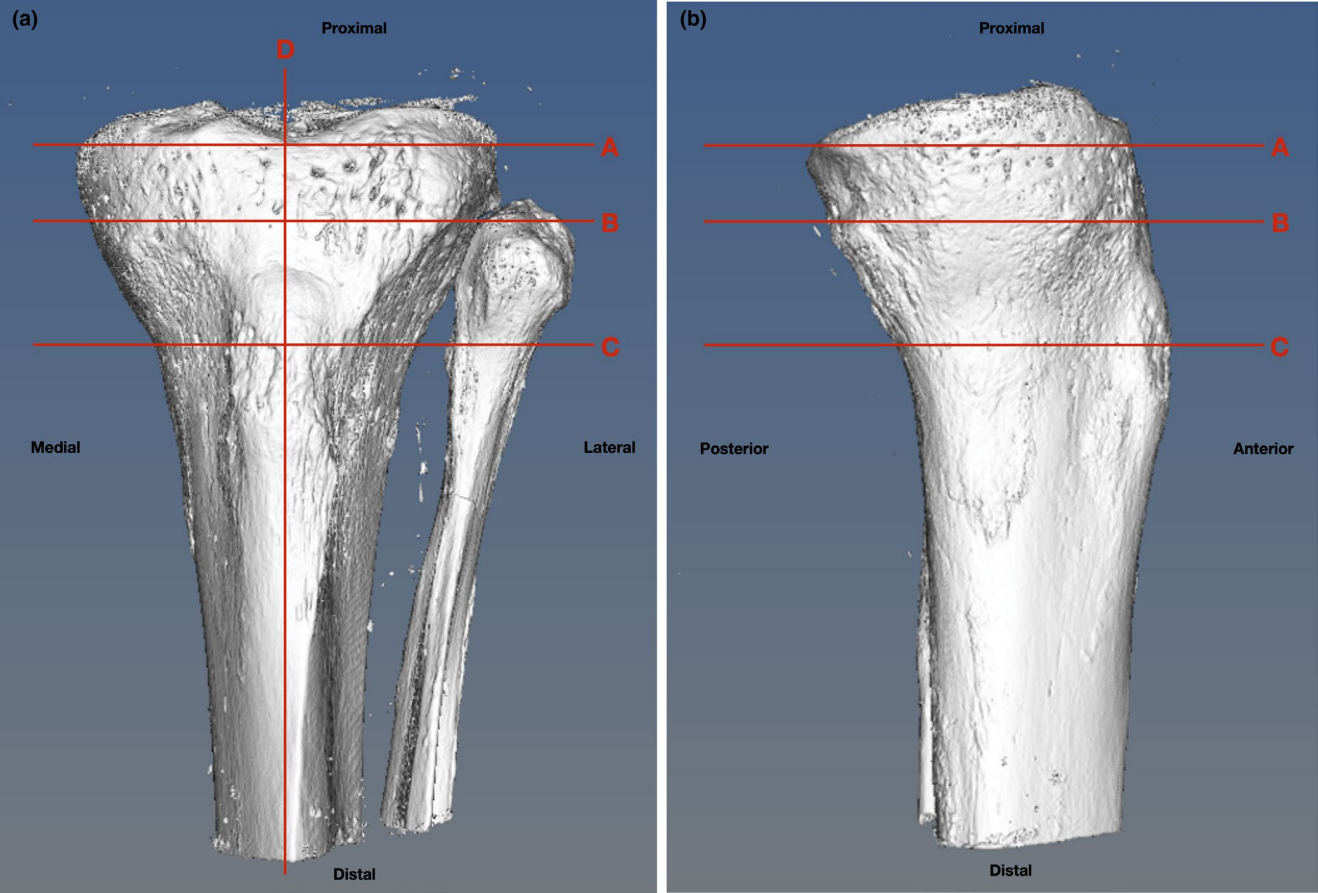
Appearance of the virtual tibial plateau following reconstruction of DICOM output using MicroView. (a) Antero‐posterior (AP) view of the left tibial plateau from specimen 5 (female, Age 76 years, cause of death: heart failure). (b) Medio‐lateral (ML) view of the same specimen. Letters indicate sections at which ROIs were measured and positioned (see Figure 2). Image taken during reconstruction on 08/03/19

Scans were loaded onto the open‐source MicroView 3D Image Viewer and Analysis Tool (Parallax Innovations Inc.). Individual slices were reconstructed into a 3D model of each tibial plateau (see Figure 1). We chose to compare sub‐entheseal trabecular properties at the Segond site with other entheses across the tibial plateau and fibular head. Spheres of 5 mm diameter were constructed to define the portion of the bone to be analysed. These ‘regions of interest’ (ROIs) were positioned to underlie ten entheseal or compression sites across the tibial plateau (numbered below). Precise locations below refer to the centre point of the virtual biopsy ROI. They were chosen based on preliminary measurements of where the sub‐entheseal trabecular bone appeared to have the highest volume fraction at each site.

1. Anterior cruciate ligament (ACL) insertion—50% of the medial–lateral (ML) axis of the insertion (section ‘D’ in Figure 1a), 25% along anterior–posterior (AP) axis from the anterior‐most point of the ACL insertion.
2. Posterior cruciate ligament (PCL) insertion—50% of the ML axis (section ‘D’ in Figure 1a) at the posterior/inferior‐most insertion point of the PCL.
3. Patellar tendon (PT) insertion—50% of the ML axis of the tibial tuberosity, 77.5% the superior‐inferior distance along the PT insertion (section ‘C’ in Figure 1b).
4. Medial tibial condyle (MTC)—16% of ML axis of the tibial plateau from the medial edge, 55% of AP axis (section ‘A’ in Figure 1a) from anterior edge.
5. Lateral tibial condyle (LTC)—10% of ML axis of the tibial plateau from the lateral edge, 65% of AP axis (section ‘A’ in Figure 1a) from anterior edge.
6. Lateral collateral ligament (LCL) insertion—50% of the distance across the ML width of the proximal‐most insertion point of the LCL (section ‘B’ in Figure 1a).
7. Segond site α (Sα)—50% of the AP distance from Gerdy's tubercle to the posterior aspect of the fibular head (section ‘B’ in Figure 1a), 7.8 mm from the tibial plateau in the proximal–distal plane. Vertical depth from plateau chosen based on previous literature which showed the mean distance of the midpoint of the fracture to the tibial plateau to be 7.8 ± 2.7 mm (Shaikh *et al*. 2017).
8. Segond site β (Sβ)—2.5 mm anterior to Sα.
9. Segond site γ (Sγ)—2.5 mm posterior to Sα.
10. Iliotibial band (ITB) insertion—same vertical depth as (7), centred 50% of the ML width of Gerdy's tubercle.

**FIGURE 2:**
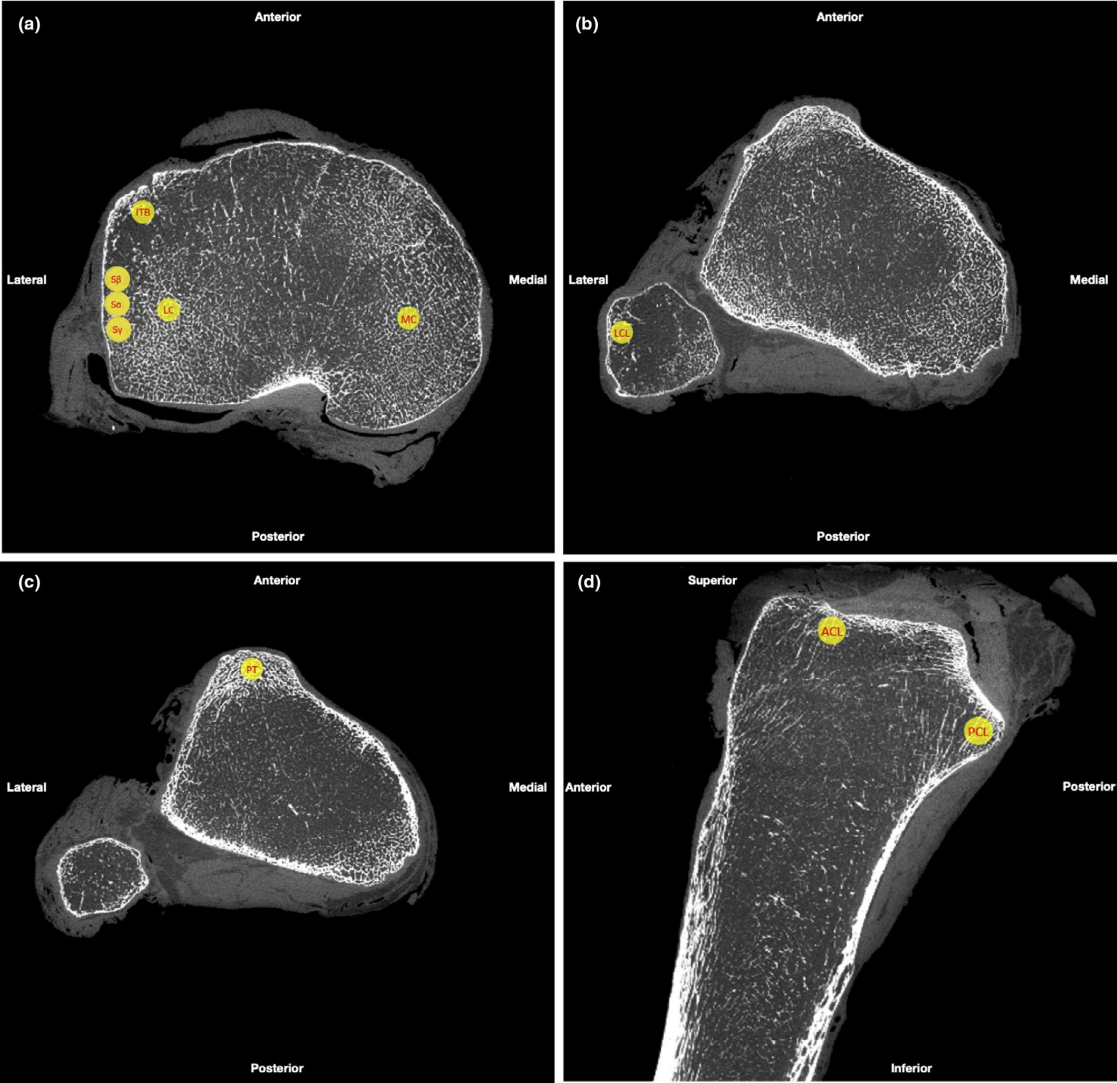
μCT sections of a reconstructed tibial plateau showing the location of each ROI. (a) Shows section ‘A’ from Figure 1—an axial view of the tibial plateau—including the ROI for MC, LC, ITB, and the Segond site. (b) Shows section ‘B’ from Figure 1—a more distal axial view of the plateau, including the head of fibula—the ROI for LCL. (c) Shows section ‘C’ from Figure 1—a more distal axial view of the plateau at the level of the tibial tuberosity—the ROI for PT. (d) Shows section ‘D’ from Figure 1—a midline sagittal view of the tibia—including the ROI for ACL and PCL. μCT images taken from Tibia 5 (left tibia, female, age 76 years, cause of death: heart failure). Image taken during reconstruction on 08/03/19

The relative locations of the ROIs detailed above are shown in Figure 2. The medial collateral ligament could not be included as its insertion varied between tibiae and thus could not be reliably found using the methodology above. The medial and lateral condyles represent compression zones: included as data to add insight into the relationship between compression force and bone volume. The Segond fracture has been stated as having an average length of 10 mm (Shaikh *et al*. 2017). Due to the 5 mm diameter sphere which defined the portion being measured, 3 ROIs were used to sample the full diameter of the Segond site and to map any variations that might be across this region: Sα, Sβ and Sγ.

Using MicroView software, BV/TV was measured at each ROI. The value represented the fraction of the ROI occupied by trabecular bone. A value of 0 would represent thin air, and a value towards 1 would be found in near‐solid cortical bone. At each ROI, a total of five replicate measurements were taken by moving the measuring tool 1 mm from the calculated centre point in four opposing directions on the cortical axis—for example, at the ACL site, the repeat measurements were taken 1 mm anterior, posterior, medial and lateral from the calculated centre point. Crucially, cortical bone was avoided during repeat measurements by keeping depth in relation to the cortex constant for each repeat measurement. This was necessary so that the higher BV/TV of cortical bone did not inflate the measurement of bone volume fraction of the underlying trabecular bone.

### 2.4 Statistical analysis

Five replicate BV/TV measurements were obtained using MicroView for each ROI in each specimen. The means of the replicates represent 10 ROI values (mean BV/TV) for each specimen. Descriptive statistics and analyses were performed on these values as described.

Univariate repeated‐measurements ANOVA (ROI = within‐subject variable) using the Greenhouse–Geisser correction for sphericity was performed using IBM® SPSS Statistics v.25 and Microsoft Excel to evaluate differences between ROIs. Normality of residuals was evaluated using the D'Agostino–Pearson test (GraphPad Prism 8.4.2). Statistical results presented are from log‐transformed data for all 10 ROIs (*n* = 200) and a condensed dataset (*n* = 160) in which Sα, Sβ or Sγ were replaced with a single value (SEG) equalling the mean of the three separate values. Pairwise within‐subject contrasts between ROIs and either Sα or SEG values were performed with a Bonferroni adjustment.

## 3 RESULTS

Mean BV/TV values for each ROI are shown in Table 1. In 15 of the 20 tibiae, the lowest intra‐specimen mean BV/TV was found at the Segond site (range = 0.092–0.262, recorded at either Sα, Sβ or Sγ). In the remaining 5 tibiae, the lowest intra‐specimen mean BV/TV value was found at either the LCL (0.128 and 0.171), ITB (0.196) or ACL (0.171 and 0.259). The highest BV/TV was found in 12 tibiae at the PT (range = 0.299–0.655), 6 at the MTC (range = 0.369–0.595) and 2 at the LTC (0.366 and 0.400). In addition to these intra‐specimen differences, tibiae also varied in their mean specimen BV/TV (range = 0.185–0.363; mean = 0.271).

**TABLE 1:** Mean BV/TV values at each ROI in 20 tibiae

Tibia	Sex	Side	Sα	Sβ	Sγ	ACL	LCL	ITB	PCL	LTC	MTC	PT	Mean specimen BV/TV	SEG^a^
A	F	R	0.179	0.161	0.212	0.186	0.189	0.205	0.238	0.307	0.315	0.338	0.233	0.184
B	F	R	0.166	0.166	0.172	0.227	0.209	0.267	0.254	0.283	0.426	0.494	0.266	0.168
C	F	L	0.207	0.210	0.209	0.245	0.316	0.286	0.269	0.366	0.303	0.309	0.272	0.209
D	F	L	0.126	0.126	0.140	0.165	0.143	0.176	0.158	0.246	0.274	0.299	0.185	0.131
E	F	L	0.182	0.195	0.206	0.247	0.171	0.239	0.254	0.447	0.542	0.273	0.276	0.194
F	F	L	0.199	0.191	0.177	0.263	0.128	0.234	0.298	0.294	0.299	0.393	0.248	0.189
G	F	L	0.241	0.208	0.242	0.247	0.236	0.196	0.340	0.241	0.534	0.393	0.288	0.230
H	F	L	0.116	0.121	0.092	0.214	0.182	0.183	0.246	0.223	0.380	0.426	0.218	0.110
I	F	R	0.179	0.160	0.167	0.214	0.214	0.237	0.295	0.400	0.386	0.376	0.263	0.168
J	F	L	0.203	0.190	0.201	0.194	0.310	0.214	0.354	0.360	0.588	0.422	0.303	0.198
K	F	R	0.264	0.262	0.277	0.287	0.334	0.341	0.384	0.286	0.367	0.461	0.326	0.268
L	F	L	0.165	0.169	0.169	0.226	0.181	0.246	0.285	0.253	0.355	0.427	0.247	0.167
M	F	L	0.185	0.172	0.178	0.171	0.188	0.208	0.220	0.234	0.369	0.339	0.226	0.178
N	F	R	0.211	0.172	0.168	0.213	0.226	0.298	0.246	0.281	0.364	0.433	0.261	0.183
O	F	L	0.212	0.226	0.228	0.269	0.307	0.374	0.317	0.294	0.431	0.655	0.331	0.222
P	M	L	0.215	0.238	0.201	0.216	0.315	0.422	0.235	0.276	0.415	0.479	0.301	0.218
Q	M	R	0.158	0.157	0.171	0.204	0.212	0.213	0.230	0.238	0.327	0.384	0.229	0.162
R	M	L	0.222	0.219	0.193	0.312	0.272	0.211	0.305	0.430	0.424	0.460	0.305	0.211
S	M	R	0.284	0.309	0.259	0.259	0.275	0.390	0.362	0.392	0.595	0.505	0.363	0.284
T	M	L	0.184	0.194	0.190	0.229	0.237	0.334	0.270	0.262	0.483	0.430	0.281	0.189
Pooled mean BV/TV			0.195	0.192	0.193	0.229	0.232	0.264	0.278	0.306	0.409	0.415	0.271	0.193
*SD* of mean BV/TV values			0.041	0.045	0.041	0.038	0.062	0.073	0.055	0.069	0.096	0.086	0.043	0.041

^a^ SEG = $\frac{\sum \left(S\alpha ;S\beta ;S\gamma \right)}{3}$.

Pooled mean BV/TV values (mean of ROI values from all 20 tibiae) at the Segond site (Sα, Sβ or Sγ) was the lowest of all the ROIs at 0.195, 0.192 and 0.193, respectively. This suggests that despite the inter‐ and intra‐specimen variation in mean BV/TV, the Segond site tends to have a lower trabecular bone volume fraction than entheseal sites elsewhere on the tibia.

**FIGURE 3:**
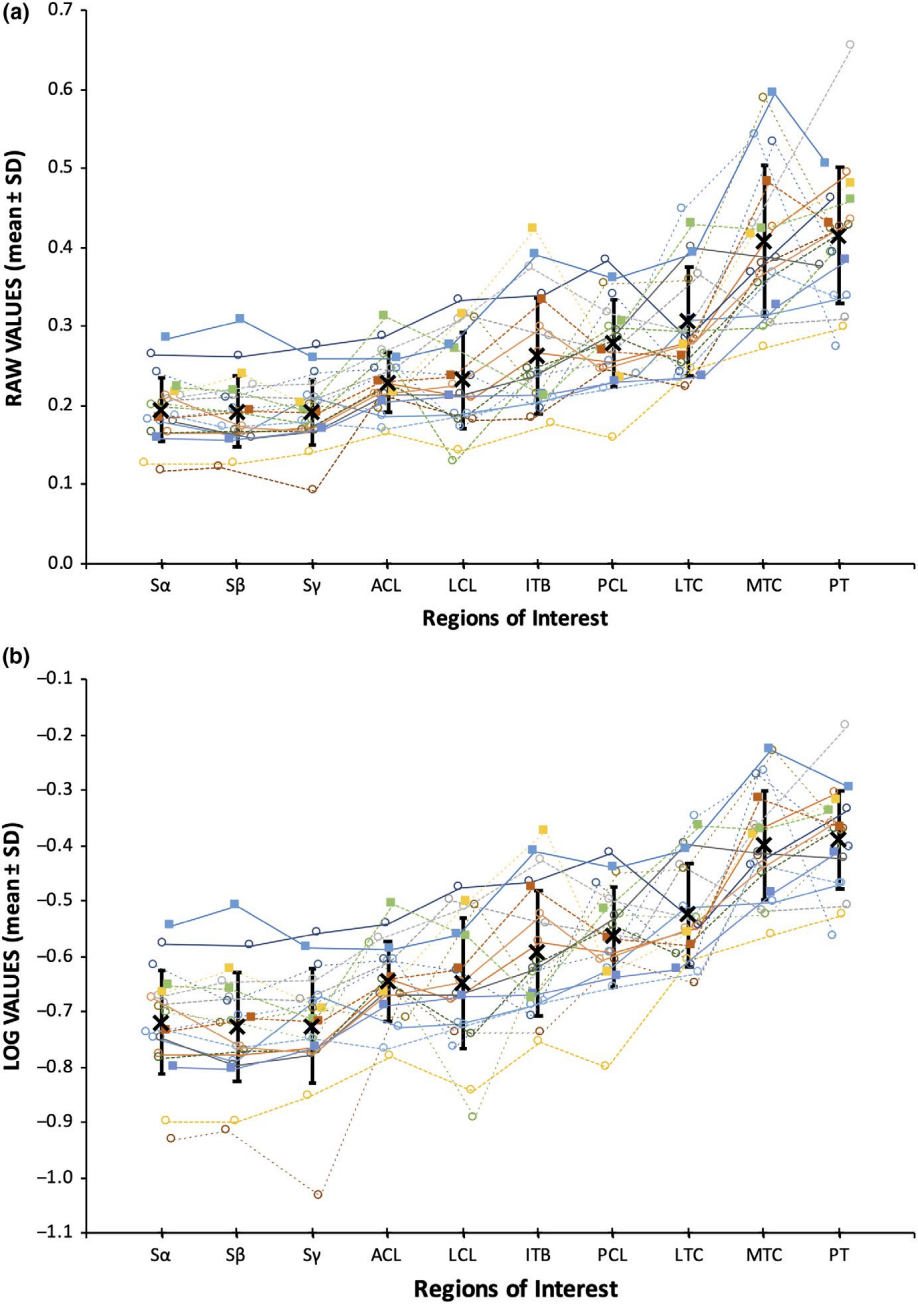
Graph showing distribution of mean BV/TV and pooled mean BV/TV data across each regions of interest. (a) Mean BV/TV data from 20 subjects for 10 different loci on the tibia. Data from a single tibia are connected by coloured lines. Filled squares: male tibiae. Open circles: female tibiae. Solid lines: right‐sided. Dashed lines: left‐sided. Pooled mean BV/TV ± *SD* shown in bold. (b) Logged mean BV/TV data from the same complete dataset

Mean BV/TV and pooled mean BV/TV data are displayed graphically in Figure 3a and logged mean BV/TV data in Figure 3b. Distribution of residuals (*n* = 200) from repeated‐measures ANOVA of non‐logged data deviated from normality (D'Agostino–Pearson test, K2 = 23.37, *p* < 0.0001) and so analysis was performed on logged data (K2 = 6.67, *p* = 0.0356). This revealed a clear difference between ROIs (*F*_5.4,102.2_ = 64.97, *p* < 0.0001). Within‐subject contrasts to Sα yielded the following *p* values (Bonferroni‐adjusted): Sβ > 0.99; Sγ > 0.99; ACL = 0.0031; LCL = 0.029; ITB, PCL, LTC, MTC & PT < 0.0001.

Sα, Sβ and Sγ ROIs were sampled from loci in very close proximity. Not surprisingly, there was a strong correlation between these values (*ρ*_(Sα v Sβ)_ = 0.93, *ρ*_(Sα v Sγ)_ = 0.89; *ρ*_(Sβ v Sγ)_ = 0.86). Since this may have influenced residual distribution, a mean value for the Segond region (SEG) was calculated from Sα, Sβ and Sγ values, and substituted the three discrete values in a parallel analysis. The distribution of residuals from the analysis of logged values (*n* = 160) conformed well to normality (K2 = 2.49, *p* = 0.29) whereas the non‐logged data retained an apparent deviation (K2 = 12.93, *p* = 0.0016).

Repeated‐measures ANOVA of logged data with the substituted SEG values (condensed dataset) also revealed a convincing difference between ROIs (*F*_4.8,91.2_ = 53.10, *p* < 0.0001). Within‐subject contrasts to SEG yielded the following *p* values (Bonferroni‐adjusted): ACL = 0.0015; LCL = 0.013; ITB, PCL, LTC, MTC & PT < 0.0001. Figure S1a and Figure S1b shows the condensed SEG‐substituted data.

A higher incidence of Segond fractures has been reported both in men (Claes *et al*. 2014) and in right‐sided tibiae (Ferretti *et al*. 2017a). Our study was not designed to evaluate any effect of sex or side, but used an opportunity sample from cadavers available for anatomical dissection. Nevertheless, among the specimens, 13 were left‐sided (10 female and 3 male) and 7 right‐sided (5 female and 2 male). Secondary analyses to explore the effect of SEX or SIDE were performed using the repeated‐measures ANOVA (with type II sum of squares) described above with incorporation of either SEX or SIDE as a between‐subjects factor. Interpretation of the results must account for the opportunistic nature of the sample; nevertheless, our data provide no support for the existence of a difference in trabecular bone density between sexes (*F*_1,18_ = 2.24, *p* = 0.152) or sides (*F*_1,18_ = 0.345, *p* = 0.564). Neither was there evidence of an interaction between ROIs and either SEX (*F*_5.4,96.7_ = 0.64, *p* = 0.68) or SIDE (*F*_5.3,96.1_ = 0.32, *p* = 0.91). (Analyses were performed on logged values of the full data set. No differences were observed when performed on the condensed data set.) Furthermore, there was no evidence that sex or side contributed to deviations from normality of the non‐grouped data (residual distribution (*n* = 200) including SEX: K2 = 8.92, *p* = 0.0116; and including SIDE: K2 = 6.32, *p* = 0.0423).

## 4 DISCUSSION

This study shows that BV/TV at the Segond site is significantly lower than other entheses across the tibial plateau. As mentioned previously, BV/TV has been shown to correlate with tensile and torsional strength—the forces putatively responsible for avulsion fractures (Kaplan *et al*. 1985; Sarin *et al*. 1999). The lower BV/TV at the Segond site could, therefore, equate to a region of local weakness in certain individuals which predisposes them to the avulsion injury following excessive internal rotation of the knee. This ‘weakest link’ hypothesis agrees with findings that the minimum BV/TV value for a specimen gave a far higher predictive power than the average specimen BV/TV in predicting the probability of mechanical failure of trabecular bone (Nazarian *et al*. 2006). Given the complex ligamentous and capsular arrangements around the knee joint, it is reasonable to assume that several structures would be placed under strain during internal rotation. The avulsion could, therefore, arise from the trabecular bone at the Segond site being a highly susceptible locus on the tibial plateau, with other injuries accumulating sequentially following progressive increases in internal rotational force. Since avulsions elsewhere on the tibial plateau occur at a much lower incidence (Bali *et al*. 2012; Edmonds *et al*. 2015; Caggiari *et al*. 2020), it may be the case that a higher entheseal BV/TV means the weakest link lies in the substance of the ligament, causing a mid‐substance tear to be more likely than an avulsion fracture.

Segond's original work described an extreme tractional force along a ‘*fibrous band’* on the anterolateral aspect of the knee during tibial internal rotation (Segond 1879, p. 14). The low BV/TV we recorded at the Segond site challenges the hypothesis that this band represents a discrete ligament, such as the ALL, as the tension exerted focally by a single ligament would be expected to elicit a hypertrophic response in line with Frost's Mechanostat hypothesis (Frost 2000). The Mechanostat argues that trabecular networks show a homeostatic response to load, including force from both compression and tension. This would explain why our highest recorded BV/TV values were found at the MTC/LTC and PT—ROIs subject to the greatest compressive and tensile forces, respectively. The Mechanostat hypothesis would argue that a locally low BV/TV would be found in a region subject to a low force per unit area. Our data would, therefore, agree with the aforementioned reports of Segond's fibrous band being part of a broader, less discrete insertion—including fibres from the ITB and lateral joint capsule (Shaikh *et al*. 2017)—which has the effect of distributing the force from internal rotation over a larger area.

The limitations of this study include the following: (a) our donor population were all over the age of 70 years, meaning the average BV/TV was unlikely to reflect population means across all ages. A solution here would be to source younger donors or perhaps tibiae from amputee donors to infer whether the trend in BV/TV continues across all age ranges. (b) The osteoporosis status of the donors was not included in their records, and thus, we were unable to categorise subjects by those with pathological demineralisation of bone. We also cannot be certain that individuals in the study did not have any previous trauma to their knee that might have resulted in ligamentous, capsular, or bony injury that might affect these results. (c) Virtual biopsy at the Segond site relied on theoretical constructs alone, based on parameters cited in previous literature. As a result, anatomical variation of the individual specimens may have resulted in biopsies being taken from locations which were not truly represent the fracture site. Our results would be ideally validated using a longitudinal study to observe which donor subgroups are at risk of avulsion injury. This would, however, not be possible using the current methodology since μCT is limited to imaging smaller, ex vivo specimens. A first step would be to mirror the data in human subjects using a technique such as DXA imaging; however, at present, the resolution of these alternate imaging methods falls short of μCT.

## CONFLICT OF INTEREST

Authors have no conflicts of interest to declare.

## AUTHOR CONTRIBUTIONS

**William Mullins:** concept/design; acquisition of data; data analysis/interpretation; drafting of the manuscript; critical revision of the manuscript. **Daniel Oluboyede**: concept/design; acquisition of data; data analysis/interpretation. **Gavin E. Jarvis**: data analysis/interpretation; critical revision of the manuscript. **Linda Skingle**: concept/design; critical revision of the manuscript. **Ken Poole**: concept/design; critical revision of the manuscript. **Tom Turmezei**: concept/design; data analysis/interpretation; critical revision of the manuscript; approval of the article. **Cecilia Brassett**: concept/design; acquisition of data; data analysis/interpretation; critical revision of the manuscript; approval of the article.

## Supporting information

Fig S1a

Fig S1b

Fig S1‐Cap

## Data Availability

The data that support the findings of this study are available on request from the corresponding author. The data are not publicly available due to privacy or ethical restrictions.
