# Supplementary figures and images for: The Segond fracture occurs at the site of lowest sub‐entheseal trabecular bone volume fraction on the tibial plateau
Source: J Anat. 2020 Aug 8;237(6):1040–8. doi: 10.1111/joa.13282 (PMC7704226; doi:10.1111/joa.13282)

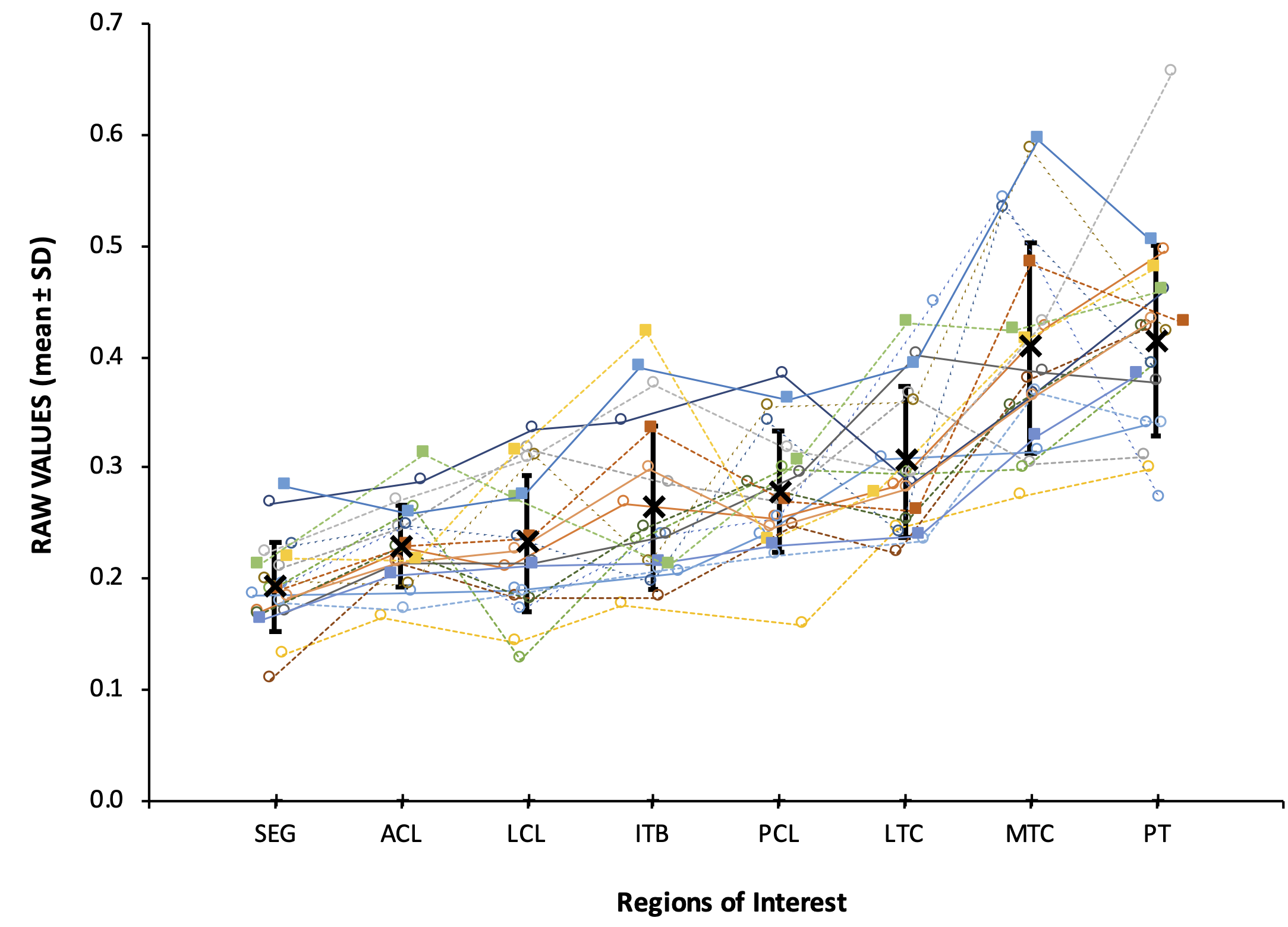

Supplement: Supplementary file 1 — Fig S1a [file JOA-237-1040-s001.tiff]

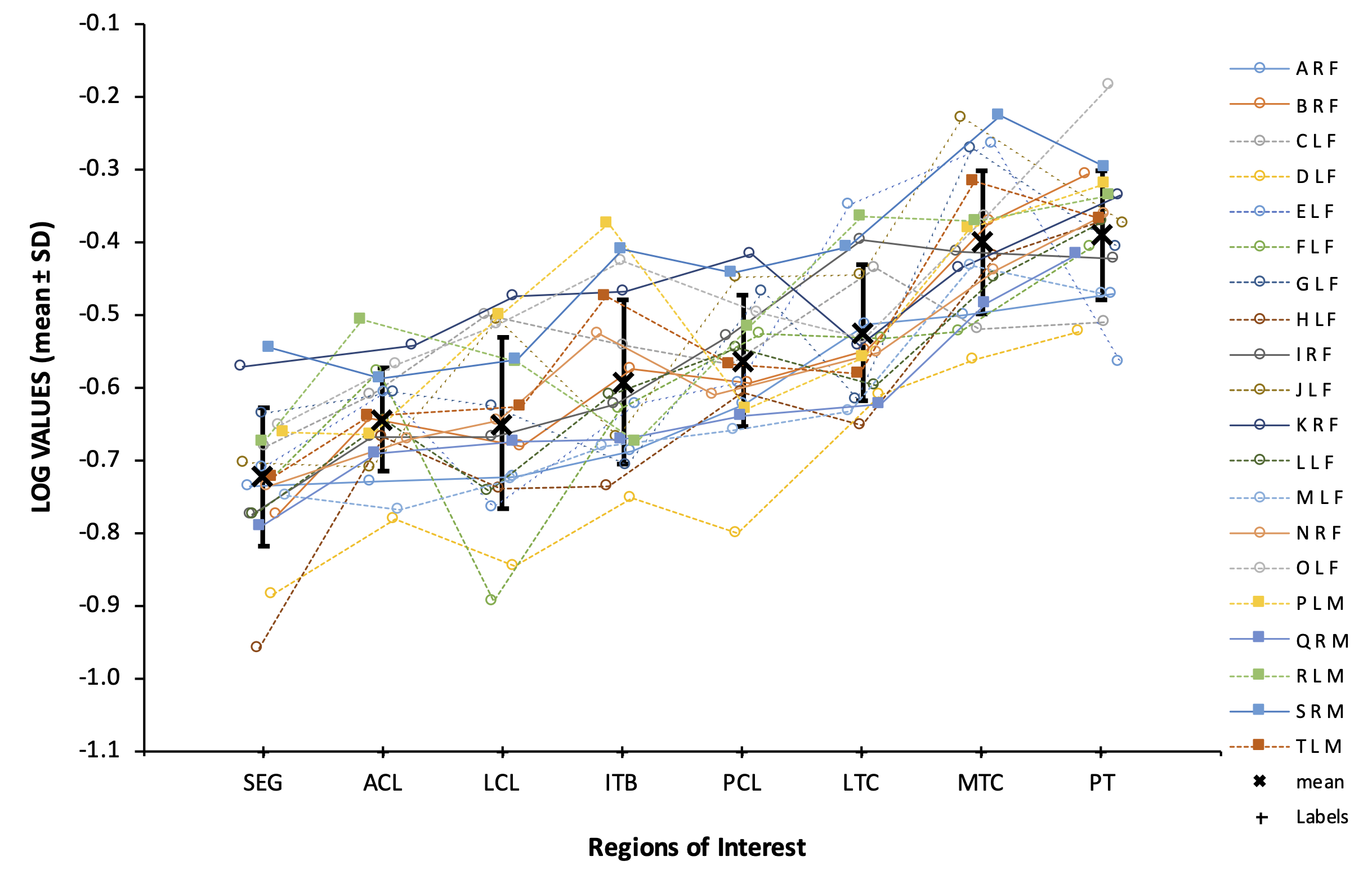

Supplement: Supplementary file 2 — Fig S1b [file JOA-237-1040-s002.tiff]
