# Supplementary material for: The Segond fracture occurs at the site of lowest sub‐entheseal trabecular bone volume fraction on the tibial plateau
Source: J Anat. 2020 Aug 8;237(6):1040–8. doi: 10.1111/joa.13282 (PMC7704226; doi:10.1111/joa.13282)
Supplement: Supplementary file 3 — Fig S1‐Cap [file JOA-237-1040-s003.docx]

**Figure S1: Graph showing distribution of mean BV/TV and pooled mean BV/TV data from the condensed dataset.** (a) Mean BV/TV data from 20 subjects for 8 different loci on the tibia. SEG = mean of Sα, Sβ, Sγ. Data from a single tibia are connected by coloured lines. Filled squares: male tibiae. Open circles: female tibiae. Solid lines: right-sided. Dashed lines: left-sided. Pooled mean BV/TV ± SD shown in bold. (b) Logged mean BV/TV data from the same condensed dataset.
